# Supplementary material for: The impact of resection margin distance on survival and recurrence in pancreatic ductal adenocarcinoma in a retrospective cohort analysis
Source: PLoS One. 2023 Feb 17;18(2):e0281921. doi: 10.1371/journal.pone.0281921 (PMC9937496; doi:10.1371/journal.pone.0281921)
Supplement: Attachment — Submitted filename: Reviewer Comments PLOS ONE 20230105.docx [file pone.0281921.s001.docx]

**Dear Editor, dear Reviewers,**

Thank you very much for the constructive feedback. We are happy to report that we agreed with most of the comments and changed the manuscript accordingly. In our opinion, the changes made based on your comments have greatly improved and strengthened the manuscript. For the few points where we disagreed, or perhaps implemented the changes slightly differently than intended, please refer to the point-by-point responses below.

**Verena Uslar**

For all authors

**Reviewer #1:**

1. In the introduction, the authors report 5-year survival for resected pancreatic cancer followed by adjuvant therapy as 17%. Would suggest a range especially that they cited the PRODIGE trial where the 5-year survival for the FOLFIRINOX arm was higher than 17%

=> Yes, we agree in selected patients with early and localized PDAC under mFOLFIRINOX the reported 5 year survival is 43.2 % compared to 31.4 % with gemcitabine. We added a paragraph with regards to this in the discussion section. However, when our paper was forwarded for publication the final analysis and updated 5 year survival outcome of the PRODIGE 24/CCTG PA6 Trial hadn’t been published. The major limitations of this trial included the inability to capture the patients who were unable to recover adequately from the operation to receive adjuvant therapy and the inability to widely apply this effective but toxic regimen to all PDAC patients because the study was highly selective. Data from the SWOG S1505 phase II trial demonstrated that median overall survival was strikingly lower than the 54.4 months seen in the PRODIGE Trial. Thus, this surely doesn’t reflect the real world data. For instance, the general 5-year survival in the United States is 11% (between 2011-2017- *adapted from the American Cancer Society's (ACS) publication,* Cancer Facts & Figures 2022, *accessed December 2022*), in Germany 10 % (2017/2018- *adapted from* [www.krebsdaten.de/pancreas](http://www.krebsdaten.de/pancreas), *accessed December 2022*). We hope that the ALLIANCE A021806 Trial will bring more light to this matter. But still many unanswered questions remain.

2. With more centers moving to neoadjuvant therapy for all stages of pancreatic cancer, it might be helpful to have this analysis done in this group of patients. That’s said, this paper is still valuable to patients with upfront resection.

=> In our cohort, there were only a few patients (n=5) who underwent neoadjuvant therapy. Therefore, we excluded this group in our study since there was no relevant data available to use in any kind of meaningful sample size calculation. Furthermore, the interpretation of such results regarding resection margin-clearance should be done with appropriate caution. Nevertheless, we fully agree that neoadjuvant therapy will (maybe) be a standard treatment in the future. Nonetheless, currently recommended guidelines for neoadjuvant therapy only exist for borderline-resectable and/or locally-advanced disease. We added a paragraph with regards to this in the discussion and we hope that the ALLIANCE A021806 Trial will bring more light to this matter. As we await a formalized report of ongoing trials, the existing findings point to the premise that the use of neoadjuvant therapy may be beneficial for patients with resectable PDAC.

3. 30 day mortality of 10% is a bit worrisome. Can the authors provide why it’s higher than expected.

=> We are sorry for this mistake. This figure refers to all patients who were treated in our clinic and thus includes patients who were inoperable. We have changed the figure to refer to the finally analyzed collective. That was 2 out of 108. Thank you for noticing that. We have amended the sentence in the results accordingly.

4. R0 of 28% is low but not surprising in upfront resected patients. Another reason why neoadjuvant should be given and why it would be interesting to do this study in this group of patients.

=> according to UICC classification and national guidelines R0, (i.e 0mm rule) was 75 % (81 out of 108) and R1 25%. But when RM >1mm rule as R0 is applied most resections are indeed R1 as previously reported. We added a sentence with regards to this in the respective section and changed the table to reflect the correct resection margin categorization.

5. Metastatic patients should not be included in this analysis. Margin status is irrelevant in patients who already have metastatic disease

=> We agree with your point. We included these patients in our cohort, since they were detected intraoperatively and resectable in the era of tailored medicine. We are though aware, that in systemic disease (like most PDAC) margin status is irrelevant.

6. I do understand why the authors do not want to use p value but comparing two confidence intervals is another way of doing inferential statistics. In addition, choosing 12 months as clinically relevant cut off for survival analysis is arbitrary and not based on any scientific merit. Since when doubling survival in cancer is the cut off for clinically relevant effect? That’s too ambitious. Therefore, saying the there is no OS difference between CRM>1mm and CRM<1mm (29.8 months vs 18.6 months) is not convincing. It’s true the CI overlap but this may be related to underpower due to small sample size. I would say if this were a real difference, an improvement of survival by about 11 months in pancreas cancer is a big deal.

=> First of all, thank you for not insisting on the p-values, which in our opinion do not always reflect the clinical relevance. Also, with regards to inferential statistics, we appreciate your remark and we changed the wording accordingly. You are of course right in saying that 12 months is relatively arbitrary. We agree, that especially in pancreatic cancer an improvement of survival by about 11 months is high. The difficulty in this exploratory approach was to implement a cut-off which reflects both a clinical relevance but also the appropriate caution with regards to sample size and so on. We changed the wording accordingly in the statistics section and the results, and added half a sentence in the discussion.

7. How do the authors explain that median survival of <1mm is better than >1mm and <2mm in table 2 and 3. What is the n for each group?

=> the group size was n = 22 with RM < 1mm and n = 10 for ≥1mm and < 2mm. This information was added in the result section. Based on this sample size one should be very cautious in saying, that this 2 and 3 months respectively, reflect a real difference between those groups.

8. How do the author explain that the recurrence for patients without margin involvement is 41% higher than 15% for positive posterior margin

=> We are of the opinion that 1.), given the small sample size for patients with positive posterior margin involvement in our study cohort, our findings must be interpreted with caution, e.g. institutional bias, 2.) our results reflect the already known data, that even in these so called R0 resections most patients may have had a systemic occult disease. This supports the biologic heterogeneity of this tumor, suggesting multifactorial and simultaneous factors affecting long term survival and recurrence after resection and not only resection margin status. Lastly, the local radicality of the posterior margin is limited by anatomical structures compared to other margins like medial margin. Thus, directly limiting the possibility of extended resections to improve survival of this deadly cancer.

9. The authors are very conclusive about their findings which is not valid based on a small study prone to false negative findings. Especially when they conclude that there is no OS difference between CRM>1mm and CRM<1mm (29.8 months vs 18.6 months). 11 months difference is clinically relevant difference even if CI overlap.

=> We strongly agree that based on the small sample size analyzed in each subgroup, we should be very careful with the interpretation of our results. We therefore changed our wording accordingly in the discussion and conclusion. We hope that this now reflects the caution with which we ourselves view this data. Zhang et al. (41) also analyzed 84 patients and showed he importance of the medial resection margin. Furthermore, Gebauer et al. (15) analyzed 118 patients and showed that wider RM clearance (2 mm or more) improved OS compared with a clearance of less than 2 mm. Therefore, we recommend in our discussion, that a matched analysis in a prospective multicentric setting should be conducted to confirm our findings.

**Reviewer #2:**

Dear authors, first of all congratulations to a nice work. I only have minor additional questions. My questions to you are the following:

1. Do you have preoperative Tumormarker-levels (especially CA19-9) and did you try to include it into your work? Especially for the multiple logistic regression analysis on risk of cancer recurrence it might be another interesting factor.

=> Thank you very much for this kind assessment, and the very constructive feedback. We refrained including tumor marker like CA 19-9 from our analysis since this analysis would in our view completely change the intention and the structure of this paper. We hope for your understanding.

2. How many patients did recieve adjuvant chemotherapy with nab-Paclitaxel and Gemcitabin and was it perfomed within a clinical trial? To my knowledge the indication is in unresectabel or metastatic pancreatic cancer as it had no overall survival benefit to Gemcitabin-monotherapy in the adjuvant setting (DOI: 10.1200/JCO.2019.37.15_suppl.4000 Journal of Clinical Oncology 37, no. 15_suppl (May 20, 2019) 4000-4000).

=> Thank you very much for noticing this mistake. After your comment we analyzed our prospectively maintained database and found 2 patients incorrectly assigned with adjuvant nab-paclitaxel and gemcitabin. These two cases were first treated with gemcitabin und developed in the course of 6-12 months metastasis. Thereafter they received gemcitabin plus nab-paclitaxel. We corrected this accordingly.

3. What imaging technique did you use to evaluate resectability?

=> Both CT and MRI/MRCP scan were used during discussion in the multidisciplinary tumor board (Bae, Jae Seok, et al. "Prediction of residual tumor and overall survival after first-line surgery in patients with pancreatic ductal adenocarcinoma using preoperative magnetic resonance imaging findings." *Acta Radiologica* 63.4 (2022): 435-446., Somers, Inne, and Shandra Bipat. "Contrast-enhanced CT in determining resectability in patients with pancreatic carcinoma: a meta-analysis of the positive predictive values of CT." *European radiology* 27.8 (2017): 3408-3435.). Very rarely was endoscopic ultrasonography used to formulate the final decision. We added this information to the methods section.

4. Based on your findings, would you recommed MRI imaging for the tumours who are likely to involve the medial resection margin for a better preoperative assessment? Thank you for your answers. I am looking forward to see a multicentric clinical trial to confirm the findings on impact of different resection margins distance or clearance on recurrence and survival.

=> Thank you very much for your kind remarks. Based on the accumulating experience in our institution, both CT and MRI are useful for preoperative assessment, if conducted in a correct manner- i.e. thin-slice imaging. Moreover, no imaging can yet predict the definite R0 resection at the medial margin. We have not included anything about this in the manuscript currently because it is an institutional opinion and not evidence based.
